# High Human Papillomavirus DNA loads in Inflammatory Middle Ear Diseases

**DOI:** 10.3390/pathogens9030224

**Published:** 2020-03-18

**Authors:** Nicola Malagutti, John Charles Rotondo, Luca Cerritelli, Claudio Melchiorri, Monica De Mattei, Rita Selvatici, Lucia Oton-Gonzalez, Francesco Stomeo, Manuela Mazzoli, Michela Borin, Beatrice Mores, Andrea Ciorba, Mauro Tognon, Stefano Pelucchi, Fernanda Martini

**Affiliations:** 1 ENT Department, University Hospital of Ferrara, 44121 Ferrara, Italy; nicolamalagutti@gmail.com (N.M.); luca.cerritelli.bo@gmail.com (L.C.); claudio.melchiorri2@gmail.com (C.M.); francesco.stomeo@unife.it (F.S.); mmazzoli@ospfe.it (M.M.); michelaborin1979@gmail.com (M.B.); beatrice.mores@student.unife.it (B.M.); stefano.pelucchi@unife.it (S.P.); 2 Department of Morphology, Surgery and Experimental Medicine, University of Ferrara, 44121 Ferrara, Italy; rtnjnc@unife.it (J.C.R.); monica.demattei@unife.it (M.D.M.); lucia.otongonzalez@unife.it (L.O.-G.); tgm@unife.it (M.T.); 3 Department of Medical Sciences, University of Ferrara, 44121 Ferrara, Italy; rita.selvatici@unife.it

**Keywords:** HPV, virus, infection, viral DNA load, inflammation, middle ear, chronic otitis media

## Abstract

**Background**. Previous studies reported human papillomaviruses (HPVs) in middle ear tumors, whereas these viruses have been poorly investigated in chronic inflammatory middle ear diseases. We investigated HPVs in non-tumor middle ear diseases, including chronic otitis media (COM). **Methods**. COM specimens (n = 52), including chronic suppurative otitis media (CSOM) (n =38) and cholesteatoma (COMC) (n = 14), as well as normal middle ear (NME) specimens (n = 56) were analyzed. HPV sequences and DNA loads were analyzed by quantitative-PCR. HPV genotyping was performed by direct sequencing. **Results**. HPV DNA was detected in 23% (12/52) of COM and in 30.4% (17/56) of NME (*p* > 0.05). Specifically, HPV DNA sequences were found in 26.3% (10/38) of CSOM and in 14.3% (2/14) of COMC (*p* > 0.05). Interestingly, the HPV DNA load was higher in COMC (mean 7.47 copy/cell) than in CSOM (mean 1.02 copy/cell) and NME (mean 1.18 copy/cell) (P = 0.03 and P = 0.017 versus CSOM and NME, respectively). HPV16 and HPV18 were the main genotypes detected in COMC, CSOM and NME. **Conclusions**. These data suggest that HPV may infect the middle ear mucosa, whereas HPV-positive COMCs are associated with higher viral DNA loads as compared to NME.

## 1. Introduction

Human papillomavirus (HPV) infection is often associated with benign diseases and malignant tumors affecting the upper respiratory tract, including respiratory papillomatosis and oropharyngeal cancers [1,2]. Several studies also reported HPV involvement in the development of middle ear squamous cell carcinoma [3,4,5,6,7,8,9]. Few studies are currently available for HPV in non-tumor middle ear diseases, such as chronic otitis media (COM), including chronic suppurative otitis media (CSOM) and chronic otitis media with cholesteatoma (COMC) [3,4,5,6,7,8,9,10]. 

CSOM is a middle ear disease that relies on chronic inflammation. Different pro-inflammatory cytokines, such as TNF-α, IL-1β, IFN-γ and IL-6, have been found to be up-regulated in the middle ear mucosa sampled from CSOM patients [11]. However, the etiology of CSOM remains to be determined. The relationship between HPV infection and inflammation has been previously reported [12]. It has been shown that persistent infection with high-risk HPVs leads to an increase in pro-inflammatory cytokines, including IL-6, TNF-α and MIP-1α [13]. In addition, high-risk HPV type 16 (HPV16) is able to increase the expression of cyclooxygenase-2 (COX-2), a key enzyme in the synthesis of prostaglandins, which are important mediators of inflammation [14,15]. Until now, only a single study has reported HPV DNA sequences in CSOM, whereby different HPV genotypes, including HPV16, HPV18 and HPV6, have been detected in 30.7% of CSOM [4]. 

COMC is a form of expanding growth consisting of keratinizing squamous cell epithelium [16]. There is great interest in the etiopathogenesis of HPV-associated cholesteatoma because HPV commonly infects the stratified epithelium [17,18]. However, conflicting data have been reported for HPV in COMC [10,19,20,21]. HPV sequences have been detected in COMC with different prevalence, ranging from 3% to 70% [10,19,20,21]. Moreover, no specific HPV genotypes have been associated with COMC, as high- and low-risk HPVs, such as HPV16, HPV18 and HPV6 and HPV11, have been detected [10,19,20,21]. 

There is emerging evidence that HPV infection can occur in different anatomical sites. Since HPV infects epithelia [22], all anatomical sites covered with epithelial tissue are potentially exposed to HPV infection. Apart from pluristratified tissues of the cervix [23], vulva [24] and oral pharynx [25], HPV sequences have been detected in simple epithelia from several anatomical sites such as lung [26], upper respiratory tract [27], larynx [28] and nose [29]. Since the middle ear mucosa is composed of respiratory epithelium, and it is connected with the Eustachian tube to the oral and respiratory regions, HPV infection may also occur in the middle ear mucosa. However, previous studies have been mainly performed in COM and tumor samples, and no studies are currently available on HPV infection in normal middle ear mucosa. 

Therefore, in this study, HPV sequences, viral DNA load and HPV genotypes were investigated in middle ear specimens from patients affected by COM, including CSOM and COMC, as well as in normal middle ear (NME) specimens.

## 2. Results

### 2.1. HPV DNA Detection 

**Figure 1:**
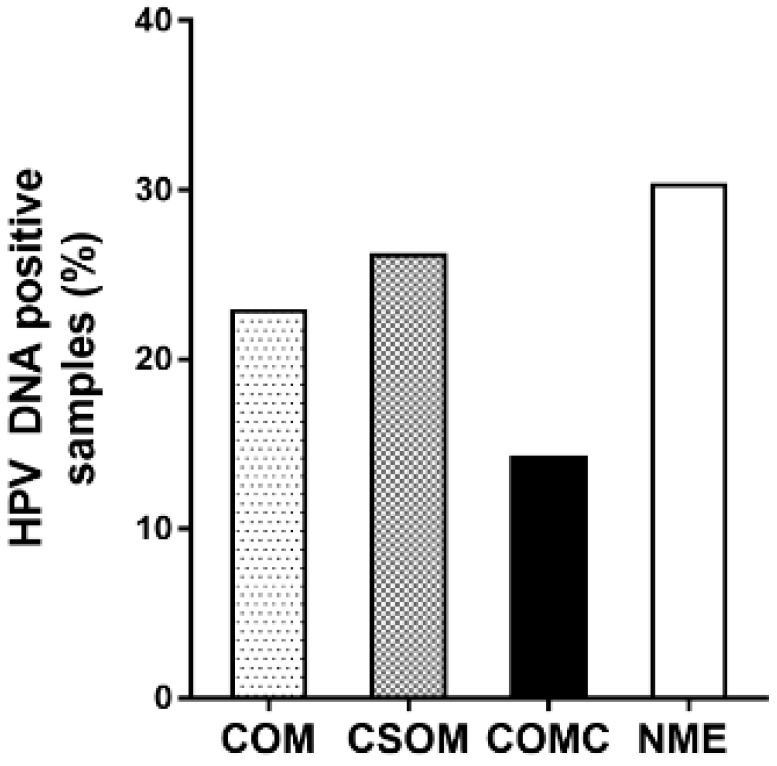
Prevalence of human papillomavirus (HPV) DNA in middle ear mucosa specimens. The presence of HPV DNA was investigated in chronic otitis media (COM) specimens (n = 52), including chronic suppurative otitis media (CSOM) (n = 38) and chronic otitis media with cholesteatoma (COMC) (n = 14) as well as normal middle ear (NME) specimens (n = 56). No statistically significant differences were observed within groups (*p* > 0.05).

HPV sequences were investigated by quantitative PCR (qPCR) in COM specimens (n = 52), including CSOM (n = 38) and COMC (n = 14), as well as in NME specimens (n = 56). Overall, HPV DNA was detected in 23% (12/52) of COM and 30.4% (17/56) of NME specimens (*p* > 0.05; Figure 1). Specifically, 26.3% (10/38) of CSOM and 14.3% (2/14) of COMC tested positive for HPV sequences (*p* > 0.05; Figure 1). 

### 2.2. HPV DNA Load 

**Figure 2:**
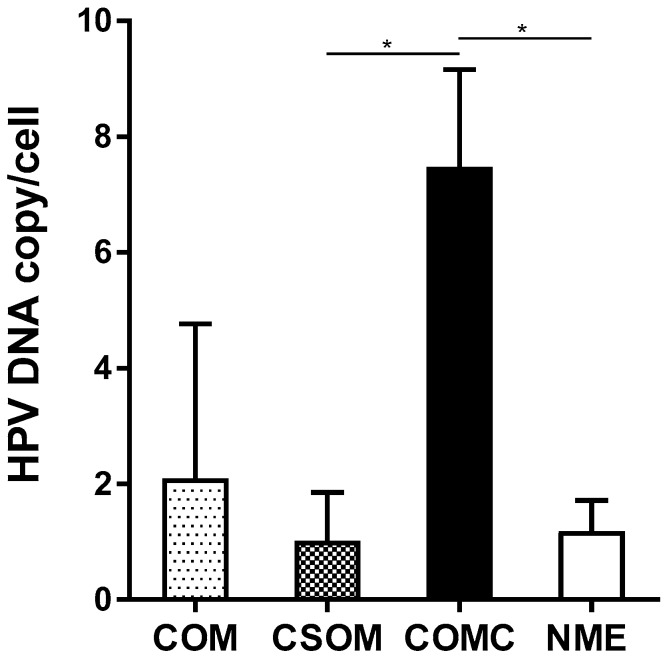
Mean HPV DNA load detected by qPCR analysis. The mean HPV DNA load (viral DNA copy/cell) was determined in HPV-positive COM specimens (n = 12), including CSOM (n = 10) and COMC (n = 2) as well as NME specimens (n = 17). Error bars represent standard error of mean. * *p* < 0.05 versus CSOM and NME.

HPV DNA load from middle ear mucosa specimens was determined by qPCR (Figure 2, Table 1). The mean of HPV DNA load was 2.09 copy/cell (range 0.01–8.67 copy/cell) in COM (n = 12) and 1.18 copy/cell (range 0.20–1.96 copy/cell) in NME (n = 17) specimens (Figure 2). 

Specifically, the mean HPV DNA load was 1.02 copy/cell (range 0.01–2.36 copy/cell) in CSOM (n = 10) and 7.47 copy/cell (range 6.28–8.67 copy/cell) in COMC (n = 2) specimens. The difference in the HPV DNA load between COMC and CSOM as well as between COMC and NME was statistically significant (P = 0.03 and P = 0.017 versus CSOM and NME, respectively, Table 1). Although the number of COMC was very limited in our sample (n = 14), it was interesting to verify that the two HPV-positive COMC samples carried a viral DNA load that was three- and four-fold higher than the highest viral DNA load detected in the HPV-positive CSOM and NME samples, respectively.

### 2.3. HPV Genotyping

HPV genotypes were determined by direct sequencing analysis in HPV-positive (n = 29) middle ear mucosa specimens. Twenty-nine qPCR products from CSOM (n = 10), COMC (n = 2) and NME (n = 17) were sequenced. DNA sequencing confirmed the presence of HPV in all specimens analyzed. HPV genotypes detected were HPV16, HPV18 and HPV11. Specifically, HPV16 genotype was present in 100% (2/2) COMC, 50% (5/10) CSOM and 52% (9/17) NME, while HPV18 genotype was detected in 20% (2/10) CSOM and 23.5% (4/17) NME. HPV11 genotype was detected in 20% (2/10) and 5.9% (1/17) CSOM and NME, respectively. The simultaneous presence of HPV16 and HPV18 genotypes was detected in 10% (1/10) and 17.6% (3/17) CSOM and NME, respectively. 

### 2.4. Association between HPV and Co-Factors

To evaluate the involvement of HPV co-factors in the etiopathogenesis of COM, a univariate analysis was performed between HPV-positive specimens and age, smoking and gender (Table 2). Results indicated that HPV prevalence was higher in CSOM patients aged ≥ 65 yrs compared to age-matched NME and to CSOM patients aged ≤ 64 yrs (P = 0.006 and P = 0.020 versus NME and CSOM, respectively; Table 2). Moreover, HPV prevalence was higher in NME specimens from smokers than in NME from non-smokers (P = 0.037; Table 2).

## 3. Discussion

In this study, HPV DNA sequences, viral DNA loads and genotypes were investigated in COM specimens, CSOM and COMC, as well as in NME specimens.

CSOM is a middle ear disease, which depends on chronic inflammation. The etiology of CSOM is largely unknown. High- and low-risk HPV sequences in 30.7% of CSOM have been previously reported [4]. Similarly, in the present study, 26% CSOM specimens tested positive for HPV, including high- and low-risk HPV genotypes (i.e., HPV16, HPV18 and HPV11). As HPV prevalence, viral DNA load and genotypes were similar in CSOM and NME specimens, no association between HPV and CSOM was found. However, a significantly higher HPV prevalence was detected in CSOM patients aged ≥ 65 yrs than in CSOM patients aged ≤ 65 yrs, suggesting that HPV infection and old age may be co-factors in the etiopathogenesis of CSOM. One explanation is that HPV infection may persist longer in the middle ear mucosa of older compared to younger individuals, as shown for HPV infection in uterine cervix [30]. Considering the already known promoting role of high-risk HPV infection in inflammation, our results suggest that high-risk HPV infection may play a role in triggering inflammation of the middle ear mucosa in the elderly, ultimately leading to the development of CSOM. Thus, high-risk HPV infection of the middle ear may explain, at least in part, in a subset of COM, the etiopathogenesis of CSOM.

COMC is a disorder of the middle ear consisting of keratinizing squamous cell epithelium. Studies of HPV in COMC reveal widely differing prevalence rates, ranging from 3% to 70% [19,31,32], with some studies reporting detection of only low-risk HPVs, HPV6 and HPV11 [3,10], and others both low- and high-risk HPVs, mainly HPV16 [3]. In our study, the HPV prevalence was 14% in COMC, and the high-risk HPV16 was the only genotype detected. Interestingly, the HPV16 DNA load was significantly higher in COMC (mean 7.47 copy/cell) compared to CSOM (mean 1.02 copy/cell) and NME (mean 1.18 copy/cell). This result may reflect the higher proliferation rate of HPV-infected cells from COMC compared to CSOM and NME, suggesting that viral DNA replication may occur in COMC. It has been reported that HPV infection leads to the expression of the viral oncoproteins E6 and E7, which stimulate cell growth and viral DNA replication [22,33,34,35,36,37]. Although no studies are available on HPV DNA load in normal and pathological middle ear tissues, our results are in agreement with previous uterine cervix investigations reporting a mean of viral DNA copy/cell ranging from 0.1 to 18 in cervical intraepithelial neoplasia and less than one viral DNA copy/cell in normal cervical tissues [37,38]. Thus, our data support and extend previous results confirming that HPV sequences can be detected in COMC. Moreover, the increased viral DNA load in COMC compared to CSOM and NME provides indirect evidence of an active infection, suggesting that HPV may play a role in the development of COMC. 

The role of HPV infection in middle ear disease onset remains to be elucidated. In this context, it should be remembered that some HPV-infected subjects, due to their genetic or immunological characteristics, are more prone/susceptible to viral activity resulting in disease, as reported for HPV-positive uterine cervix tissues [30]. It is possible that older patients, due to immune senescence, do not react in the normal way to HPV infection in the middle ear mucosa, with the result that the higher HPV DNA load favors the inflammation process and disease onset/progression.

Notably, it cannot be excluded that the presence of concomitant pathogenic agents, during HPV infection, could potentially increase host susceptibility to developing inflammatory middle ear diseases. Indeed, a number of case–control studies have reported an association between pathogenic infectious agents, such as *Staphylococcus aureus* [39,40] and *Pseudomonas aeruginosa* [41,42,43] with inflammatory middle ear diseases. Further studies that investigate viral and bacterial co-infections may clarify this issue.

The difference in HPV DNA prevalence, in available studies, could be related to (i) differences in sampling (i.e., frozen storage as in our study) versus formalin fixed [10,19,20,21]; (ii) storage conditions and (iii) method sensitivity for HPV DNA isolation and PCR detection [10,19,20]. Further studies with a larger sample size that includes cancer tissues are needed to assess the role of high-risk HPV infection in COMC.

In this study, for the first time, HPV sequences were detected in NME. Indeed, HPV sequences were found in 30% of NME, indicating that the middle ear mucosa is an additional epithelial tissue susceptible to HPV infection. High- and low-risk HPV genotypes (i.e., HPV16, HPV18 and HPV11) were detected in the middle ear mucosa. This result is in agreement with previous studies, which reported HPV infection in several normal tissues [25,44]. Although middle ear epithelium is not considered to be the primary target tissue for HPV, factors such as inflammation and smoking can trigger the development of metaplastic tissue, which is the preferred target tissue for viral infection. It is well known that metaplasia arises in the middle ear during acute and chronic events triggered by infectious agents or smoking. Accordingly, in our study HPV sequences were found to be present at higher prevalence in NME specimens from smokers than in NME from non-smokers, indicating that smoking may favor HPV middle ear infection. 

## 4. Materials and Methods 

### 4.1. Patients and Specimens

Middle ear mucosa specimens were collected from patients (n = 52) suffering from COM (mean age ± standard deviation (SD), 47.6 ± 16.2 yrs), including CSOM (n = 38) (mean age ± SD, 53.7 ± 18.8 yrs) and COMC (n = 14) (mean age ± SD, 41.3 ± 13.6 yrs). NME specimens (n = 56) (mean age ± SD, 44.2 ± 19.4 yrs) were collected from patients undergoing ear surgery for cochlear implantation or stapedoplasty. The exclusion criterion was no previous ear surgery. Middle ear mucosa specimens were collected during surgery, or by middle ear swab through a tympanic perforation with a micro-otoscopy. The study was performed in accordance with the Declaration of Helsinki (2008). Institutional Review Board (IRB) approval was obtained from University Hospital of Ferrara Ethical Committee (Authorization n. 160986, December 12, 2016). Informed written consents were obtained from patients.

### 4.2. DNA Isolation

DNA was isolated according to standard procedures [45,46]. Briefly, tissue specimens were incubated overnight with 100 ng/µL of proteinase K at 56 °C to allow tissue digestion [47]. DNA was isolated using a QIAmp DNA Blood and Tissue Extraction Kit (Qiagen, Milan, Italy) [48]. For control, DNAs were extracted together with a sample of salmon sperm DNA and a mock sample lacking DNA. After purification, DNA was quantified by spectrophotometric reading (NanoDrop 2000, Thermo Scientific). DNA suitability for PCR analysis was evaluated by amplifying the *β-globin* gene [18]. DNA samples were then stored at −80 °C until the time of the analysis.

### 4.3. Viral DNA Load Quantification

HPV DNA load was quantified by qPCR assay, using SYBR green, with the CFX96 Touch™ RT-PCR Detection System (Bio-Rad, Segrate, Milan, Italy). DNA samples were analyzed by qPCR for HPV DNA sequences using the universal primer pair GP5+/GP6+, as reported previously [49]. Briefly, 50 ng of human genomic DNA was used in 10 μL qPCR reactions, including 2x of the SsoAdvanced Universal SYBR Green Supermix, Bio-Rad (Hercules, CA, USA) and 0.5 μM of each primer. PCR thermal conditions were as follows: an initial step at 95 °C for 5 min and 40 cycles at 95 °C for 15 s and 60 °C for 30 s [49]. Each qPCR experiment was carried out using the recombinant plasmid vector containing the complete HPV16 genome (NC_001526.4), used as positive control. A standard curve was employed using 10-fold dilutions, from 10^8^ to 10 copies, of specific recombinant plasmid to calculate the viral DNA load [50]. Human *β-globin* gene was used to determine the human cell equivalents of each sample under qPCR analysis. HPV DNA load values were reported as viral copies per human cell equivalents (copy/cell). Negative controls were the two samples used during the DNA extraction (i.e., salmon sperm DNA and mock samples) and two qPCR controls, including HPV-free human DNA and a non-template control [51]. Samples were run in triplicate for each qPCR assay. Experiments were run three times by different operators. 

### 4.4. HPV Genotyping

HPV genotypes were determined by direct sequencing analysis in HPV-positive middle ear samples. qPCR amplicons were purified using the QIAquick PCR Purification Kit (Qiagen) [52]. Purified qPCR amplicons were sequenced with automated ABIPrism 3730xl Genetic Analyser (Applied Biosystems) [53]. The resulting HPV DNA sequences were BLAST versus HPV DNA belonging to different viral strains present in the National Center for Biotechnology Information (NCBI) database (http://www.ncbi.nlm.nih.gov/blast/Blast.cgi) [54]. 

### 4.5. Statistical Analysis

The prevalence of HPV DNA in CSOM, COMC and NME specimens was evaluated by a two-sided chi-square test. Viral DNA load values were analyzed with the D’Agostino−Pearson test, and means were compared with the non-parametric Kolmogorov−Smirnov test. Univariate analysis was employed to compare the features of CSOM, COMC and NME patients, such as age, gender and smoking, in association with HPV. Statistical analyses were performed using Graph Pad Prism version 5.0 for Windows (Graph Pad, La Jolla, CA, USA) [55,56]. P values < 0.05 were considered statistically significant [57]. 

## 5. Conclusions

In conclusion, this study shows that HPV sequences are present in CSOM, COMC and normal middle ear specimens. We also show that the high-risk HPV16 and HPV18 are the main genotypes detected in CSOM, COMC and NME specimens. Lastly, although HPVs have been detected in CSOM, COMC and NME with similar prevalence, high-risk HPV DNA load was higher in COMC compared to CSOM and NME. Taken together, these data indicate that CSOM and COMC epithelia and normal middle ear mucosa are target tissues for HPV infection. It remains to be assessed whether the higher HPV DNA load detected in COMC is significant for a putative pathogenic role of HPV in this middle ear disease.

## Figures and Tables

**Table 1:** Mean human papillomavirus (HPV) DNA load in middle ear mucosa specimens. revealed by qPCR analysis.

Middle Ear Mucosa Specimens	Number of Patients	Mean HPV DNA Load (Copy/Cell)	Range (Copy/Cell)
COM	12	2.09	0.01–8.67
CSOM	10	1.02	0.01–2.36
COMC	2	7.47 **^†,‡^**	6.28–8.67
NME	17	1.18	0.20–1.96

COM: chronic otitis media; CSOM: chronic suppurative otitis media; COMC: chronic otitis media with cholesteatoma; NME: normal middle ear. ^†^ P = 0.03 versus CSOM; **^‡^** P = 0.017 versus NME.

**Table 2:** Association between HPV and co-factors in CSOM, COMC and NME.

Patients	CSOM	COMC	NME
**Age (yrs)**			
≤64	5/22 (23)	1/14 (7)	16/44 (36)
≥65	10/16 (63) ^†,‡^	0/0 (0)	1/12 (8)
**Smoke**			
Yes	4/10 (40)	1/6 (17)	5/7 (71) ^§^
No	6/28 (21)	0/8 (0)	12/49 (25)
**Sex**			
M	5/20 (25)	1/6 (17)	6/20 (30)
F	5/18 (28)	0/8 (0)	11/36 (31)

^†^ P = 0.02 versus CSOM aged ≤ 64 yrs; ^‡^ P = 0.006 versus NME aged ≥ 65 yrs; ^§^ P = 0.037 versus NME non-smokers.
